# Impact of late parent–child relationship changes on parental depression: a longitudinal aging panel study

**DOI:** 10.1186/s12889-025-22516-7

**Published:** 2025-04-15

**Authors:** Sujin Kim, Yun Seo Jang, Eun-Cheol Park

**Affiliations:** 1 Department of Public Health, Graduate School, Yonsei University, Seoul, Republic of Korea; 2 Institute of Health Services Research, Yonsei University, Seoul, Republic of Korea; 3 Health Insurance Review & Assessment Service, Wonju, Republic of Korea; 4 Department of Preventive Medicine, Yonsei University College of Medicine, Seoul, Republic of Korea

**Keywords:** Parent–child relationship, Parental depression, Frequency of parent–child contact, Financial support, Mental health

## Abstract

**Background:**

The global population of older adults is increasing, and late-life depression is becoming a significant issue. A strong relationship with one’s children is a potential factor in alleviating the risk of late-life depression. This study explored the importance of parent–child relationships, including contact, meetings, and financial support, and examined their association with depressive symptoms in older parents.

**Methods:**

Data were collected from 4,476 participants who completed the Center for Epidemiological Studies Depression Scale- 10 items (CES-D 10) questionnaire from the Korean Longitudinal Study of Aging (2006–2020). Mixed-effects logistic regression analysis was conducted to evaluate the impact of parent–child relationships over time on depressive symptoms, while accounting for individual differences.

**Results:**

An active change in the parent–child relationship status (no → yes) was associated with lower depressive symptoms (men: odds ratio [OR] = 0.63, 95% confidence interval [CI] = 0.47–0.83; women: OR = 0.67, 95% CI = 0.51–0.88) than no change in relationship (no → no). Maintaining monthly contact (men: OR = 0.61, 95% CI = 0.47–0.78; women: OR = 0.64, 95% CI = 0.50–0.81), meeting 2–6 times per year (men: OR = 0.73, 95% CI = 0.56–0.95; women: OR = 0.76, 95% CI = 0.60–0.97), and financial support (men: OR = 0.70, 95% CI = 0.54–0.92; women: OR = 0.73, 95% CI = 0.57–0.93) were each associated with reduced parental depression.

**Conclusions:**

Among older parents, a transition toward a more active parent–child relationship was associated with reduced depressive symptoms. Therefore, promoting parent–child relationships must be considered an important component of mental health interventions.

**Supplementary Information:**

The online version contains supplementary material available at 10.1186/s12889-025-22516-7.

## Background

The global population of older adults is rapidly increasing, with an estimated 2.2 billion individuals aged 65 years and older by the late 2070 s [1]. Among the countries experiencing this demographic shift, South Korea is aging at the fastest rate. As of 2023, the proportion of Koreans aged 65 years and older reached 17.5%, and the country’s suicide rate among older adults remains one of the highest in the Orgainsation for Economic Cooperation and Development (OECD), raising serious social concerns [2]. Notably, late-life depression, which is closely linked to the high suicide rates among older adults, adversely impacts their quality of life and physical health [3–5]. Additionally, older adults in South Korea, particularly women, are at a higher risk of depression and suicide [6]

In East Asian Confucian cultures, filial piety, including the moral obligation of children to care for and visit their parents, has long been emphasized. Unlike Western cultures, parent–child relationships in Confucian societies often follow hierarchical norms, where children’s obedience and parents’ sacrifice are regarded as social expectations. The absence of filial piety in these cultures may lead to emotional isolation, depression, and even suicide among older adults. Similar trends have also been observed in other East Asian countries like China [7, 8]. Positive parent–child interactions can also help reduce gender differences in depressive symptoms [9].

At the same time, the South Korean society is characterized by intense educational aspirations for children and prolonged financial sacrifices by parents. Although this commitment reflects parents'sense of responsibility and aspirations for their children’s success, it can contribute to poverty among older adults, thereby further exacerbating their mental health issues [10, 11]. Notably, South Korea has the highest poverty rate among older adults in all OECD countries [2]. Under these circumstances, economic support for aging parents is often viewed as essential. For affluent families, it represents an expression of filial piety, whereas for economically disadvantaged families, it may serve as a meaningful repayment for the sacrifices endured by parents, despite financial hardships.

For older adults, emotional and material support from their children plays a critical role in promoting mental well-being [11, 12]. Frequent contact, meetings, and financial support from children provide a vital source of social support, offering psychological stability and alleviating depressive symptoms [13–15].

From a sociological perspective, parent–child relationships can be explained through the intergenerational solidarity theory across multiple dimensions. Associational solidarity refers to the frequency and patterns of interactions shared among family members. Functional solidarity focuses on the degree of help and resource exchanges, including financial, physical, and emotional support [16]. These interactions—contact, meetings, and financial support—are essential components of intergenerational solidarity and play a critical role in defining parent–child relationships. The multidimensional nature of these elements emphasizes the need for in-depth analysis and model development to better understand their influence [17]. However, existing research in Korea has either primarily focused on exploring the types or relational aspects of intergenerational solidarity [18, 19] or has approached associational and functional solidarity from a unidimensional or cross-sectional perspective, only examining factors such as the frequency of contact, meetings, and financial support [20, 21]. Despite these efforts, studies addressing how changes in the combinations and intensities of contact, meetings, and financial support, which explain parent–child relationships, influence parental depression remain limited. To address this research gap, in the present study, we analyzed parent–child relationships within the framework of the intergenerational solidarity theory. Specifically, we examined how the frequency of contact, meetings, financial support, and their combinations and changes over time are associated with depressive symptoms in older adults.

## Methods

### Data sources and samples

We utilized data from the Korean Longitudinal Study of Aging (KLoSA), a nationally representative longitudinal panel survey initiated in 2006. The KLoSA targets middle-aged and older adults aged 45 years and older, with data collected biennially. A stratified sampling method based on region and residential type was employed to recruit a target sample of 10,000 individuals, resulting in a panel of 10,254 participants. This survey aims to support the development of effective socioeconomic policies by examining the psychosocial, economic, and demographic characteristics of older adults. The survey includes comprehensive data on household background, personal attributes, family relationships, health, employment, income and consumption, assets, subjective expectations, and quality of life.

Data from the 1 st to 8 th waves of the KLoSA, conducted between 2006 and 2020, were used. The initial panel consisted of 10,254 participants recruited in 2006, and individuals without children at baseline, newly added panel members, and those with missing values for the study variables were excluded. Finally, 4,476 participants (1,719 men and 2,757 women) were included in the analysis (see Additional file 1).

### Variables

The Center of Epidemiologic Studies Depression Scale, 10-item version (CES-D 10) is a shortened version of the original CES-D scale, consisting of 10 items designed as a screening tool for depressive symptoms in epidemiological studies. While the CES-D 10 has been validated as a screening tool for depressive symptoms in older adults, the Korean version of the CES-D 10 has been confirmed as a valid tool in terms of measurement invariance [22], although its cutoff value has not been definitively established. The internal validity of the 10 items of the CES-D 10 measuring depressive symptoms ranges from 0.75 to 0.82. The Korean version of the CES-D 10 asks participants about their depressive symptoms over the past week. Each question offers four response options:"Rarely"(less than one day),"Sometimes"(one to two days),"Often"(three to four days), and"Always"(five to seven days). Responses of"Rarely"and"Sometimes"are scored as 0, while"Often"and"Always"are scored as 1. A total score of 4 or higher across the 10 items indicates the presence of depressive symptoms [23, 24].

Changes in parent–child relationships were assessed using a composite variable incorporating contact, meeting, and financial support. Relationships were classified as"present"if there was contact, meeting, or financial support; otherwise, relationships were classified as"absent."Changes were categorized into four groups: present-to-absent, present-to-present, absent-to-present, and absent-to-absent. (1) Contact with children, classified as"present"(0.13 times per month) if the average contact across children did not fall into"not at all"or"rarely in a year,"and (2) meeting with children, classified as"present"(0.13 times per month) if the average meeting frequency across children did not fall into"not at all"or"rarely in a year."Contact and meeting frequencies were measured across 10 categories for each child:"almost daily,""about once a week,""two to three times a week,""about once a month,""twice a month,""once or twice a year,""three to four times a year,""five to six times a year,""rarely in a year,"and"not at all."(3) Financial support was classified as"present"if parents received monetary support from any child. Standardized Cronbach’s Alpha values for the variables contact, meeting, and financial support were 0.82, 0.82, and 0.82, respectively. Covariates included demographic and health-related variables assessed at each wave of the analysis. The demographic characteristics comprised age, region, educational level, marital status, household income, employment status, participation in social activities, and life satisfaction. Health-related factors included smoking, alcohol consumption, physical activity, and subjective health status. Multivariate models were used to analyze all covariates, unless indicated otherwise,.

### Statistical analysis

The chi-squared test was used to compare general characteristics between the groups. A mixed-effects logistic regression model was employed to conduct a longitudinal analysis examining the association between intergenerational support and parental depression. This model accounted for both within-individual changes over time and between-individual differences by including random intercepts to control for clustering at the individual level (ID). Fixed effects were included to evaluate the impact of parent–child relationship, such as contact frequency, meetings, and financial support, on depressive symptoms. The time variable was defined as biennial waves, and repeated measures from the same individuals were identified using personal IDs. The analysis was conducted using SAS PROC GLIMMIX, applying a binomial distribution with a logit link function.

Subgroup analysis was performed to examine the relationship between changes in parent–child interactions and variables related to depression and life satisfaction, including employment status, household income, social activity participation, and physical activity. All analyses were performed in SAS (version 9.4), with *p* < 0.05 indicating significance. Results are presented as odds ratios (ORs) with 95% confidence intervals (CIs).

## Results

Table 1 presents the baseline characteristics of the study population in 2008, stratified by sex. A total of 4,476 participants (1,719 men and 2,757 women) were included in the analysis. Between 2006 and 2008, 11.1% of the men (n = 191) reported receiving no intergenerational support—contact, meetings, or financial support—during both time periods, whereas 77.5% of the men (n = 1,332) consistently received at least one form of support. Among women, 7.2% (n = 199) reported receiving no support, whereas 83.0% (n = 2,289) maintained at least one form of intergenerational support. The Chi-squared analysis revealed a significant association between parent–child relationships and parental depression, with significant differences observed in age, educational level, household income, marital status, employment status, social relations, physical activity, health status, and life satisfaction between men and women.

**Table 1:** Baseline (2008) characteristics of the study population according to the CESD- 10

			Depressive symptoms											
Variables			Male							Female				
	Total		Yes		No			Total		Yes		No		
	N	%	N	%	N	%	P Value	N	%	N	%	N	%	P Value
**Total = 4,476**	1,719	(100.0)	387	(22.5)	1,332	(77.5)		2,757	(100.0)	837	(30.4)	1,920	(69.6)	
**Changes in parent–child relationships**							** <.0001**							**<.0001**
**No → No**	**191**	**(11.1)**	**31**	**(16.2)**	**160**	**(83.8)**		**199**	**(7.2)**	**32**	**(16.1)**	**167**	**(83.9)**	
** Yes → No**	**37**	**(2.2)**	**11**	**(29.7)**	**26**	**(70.3)**		**49**	**(1.8)**	**16**	**(32.7)**	**33**	**(67.3)**	
** No → Yes**	**159**	**(9.2)**	**32**	**(20.1)**	**127**	**(79.9)**		**220**	**(8.0)**	**51**	**(23.2)**	**169**	**(76.8)**	
** Yes → Yes**	**1,332**	**(77.5)**	**313**	**(23.5)**	**1,019**	**(76.5)**		**2,289**	**(83.0)**	**738**	**(32.2)**	**1,551**	**(67.8)**	
Age							<.0001							<.0001
45–59	540	(31.4)	74	(13.7)	466	(86.3)		858	(31.1)	135	(15.7)	723	(84.3)	
60–69	506	(29.4)	106	(20.9)	400	(79.1)		794	(28.8)	220	(27.7)	574	(72.3)	
70–79	493	(28.7)	135	(27.4)	358	(72.6)		760	(27.6)	301	(39.6)	459	(60.4)	
80 ~	180	(10.5)	72	(40.0)	108	(60.0)		345	(12.5)	181	(52.5)	164	(47.5)	
Region							0.9588							0.5686
Urban city	1,210	(70.4)	272	(22.5)	938	(77.5)		1,977	(71.7)	594	(30.0)	1,383	(70.0)	
Rural area	509	(29.6)	115	(22.6)	394	(77.4)		780	(28.3)	243	(31.2)	537	(68.8)	
Education							<.0001							<.0001
Middle school or below	992	(57.7)	276	(27.8)	716	(72.2)		2,246	(81.5)	764	(34.0)	1,482	(66.0)	
High school	524	(30.5)	89	(17.0)	435	(83.0)		433	(15.7)	59	(13.6)	374	(86.4)	
College or beyond	203	(11.8)	22	(10.8)	181	(89.2)		78	(2.8)	14	(17.9)	64	(82.1)	
Marital status							<.0001							<.0001
Married	1,548	(90.1)	323	(20.9)	1225	(79.1)		1,707	(61.9)	421	(24.7)	1,286	(75.3)	
Single	171	(9.9)	64	(37.4)	107	(62.6)		1,050	(38.1)	416	(39.6)	634	(60.4)	
Household income							<.0001							<.0001
Quartile 4	517	(30.1)	73	(14.1)	444	(85.9)		758	(27.5)	168	(22.2)	590	(77.8)	
Quartile 3	446	(25.9)	77	(17.3)	369	(82.7)		634	(23.0)	145	(22.9)	489	(77.1)	
Quartile 2	430	(25.0)	115	(26.7)	315	(73.3)		681	(24.7)	222	(32.6)	459	(67.4)	
Quartile 1	326	(19.0)	122	(37.4)	204	(62.6)		684	(24.8)	302	(44.2)	382	(55.8)	
Employment status							<.0001							<.0001
Employed	832	(48.4)	95	(11.4)	737	(88.6)		679	(24.6)	132	(19.4)	547	(80.6)	
Non-employed	887	(51.6)	292	(32.9)	595	(67.1)		2,078	(75.4)	705	(33.9)	1,373	(66.1)	
Social relations							<.0001							<.0001
Yes	1,518	(88.3)	292	(19.2)	1,226	(80.8)		2,524	(91.5)	725	(28.7)	1,799	(71.3)	
No	201	(11.7)	95	(47.3)	106	(52.7)		233	(8.5)	112	(48.1)	121	(51.9)	
Physical activity							<.0001							<.0001
Yes	575	(33.4)	86	(15.0)	489	(85.0)		747	(27.1)	176	(23.6)	571	(76.4)	
No	1,144	(66.6)	301	(26.3)	843	(73.7)		2,010	(72.9)	661	(32.9)	1,349	(67.1)	
Smoking status							0.1089							<.0001
Yes	637	(37.1)	130	(20.4)	507	(79.6)		112	(4.1)	55	(49.1)	57	(50.9)	
No	1,082	(62.9)	257	(23.8)	825	(76.2)		2,645	(95.9)	782	(29.6)	1,863	(70.4)	
Alcohol intake							0.0313							<.6261
Yes	426	(24.8)	112	(26.3)	314	(73.7)		2,158	(78.3)	660	(30.6)	1,498	(69.4)	
No	1,293	(75.2)	275	(21.3)	1,018	(78.7)		599	(21.7)	177	(29.5)	422	(70.5)	
Health status							<.0001							<.0001
Healthy	1,097	(63.8)	117	(10.7)	980	(89.3)		1,486	(53.9)	238	(16.0)	1,248	(84.0)	
Unhealthy	622	(36.2)	270	(43.4)	352	(56.6)		1,271	(46.1)	599	(47.1)	672	(52.9)	
Living with children							0.0047							0.0057
Yes	797	(46.4)	155	(19.4)	642	(80.6)		1,332	(48.3)	371	(27.9)	961	(72.1)	
No	922	(53.6)	232	(25.2)	690	(74.8)		1,425	(51.7)	466	(32.7)	959	(67.3)	
Life satisfaction							<.0001							<.0001
Good (> 70)	315	(18.3)	33	(10.5)	282	(89.5)		446	(25.9)	67	(15.0)	379	(85.0)	
Normal (50 >, < = 70)	668	(38.9)	101	(15.1)	567	(84.9)		950	(34.5)	198	(20.8)	752	(79.2)	
Bad (< = 50)	736	(42.8)	253	(34.4)	483	(65.6)		1,361	(49.4)	572	(42.0)	789	(58.0)	
Before depression							<.0001							<.0001
Yes	224	(13.0)	110	(49.1)	114	(50.9)		513	(29.8)	290	(56.5)	223	(43.5)	
No	1,495	(87.0)	277	(18.5)	1,218	(81.5)		2,244	(130.5)	547	(24.4)	1,697	(75.6)	

Table 2 presents the results of the mixed-effects logistic regression analysis examining the relationship between changes in parent–child relationships and CESD- 10-D scores, after adjusting for all covariates including age, educational level, household income, marital status, employment status, social relations, physical activity, health status, and life satisfaction. Among men, compared to those with no relationship with children, those whose relationships became changed to “present” (no → yes) showed a reduced likelihood of depressive symptoms (OR = 0.63; 95% CI = 0.47–0.83). Similarly, those with maintained relationships (yes → yes) also exhibited a decreased likelihood of depressive symptoms (OR = 0.69; 95% CI = 0.53–0.88). Similar trends were observed among women. Those whose relationships became “present: (no → yes) had a reduced likelihood of depressive symptoms (OR = 0.67; 95% CI = 0.51–0.88), and those who maintained relationships (yes → yes) also showed a reduction in depressive symptoms (OR = 0.73; 95% CI = 0.57–0.92).

**Table 2:** Results of Mixed-Effects Logistic Regression Analysis factor associated with depressive symptoms in 2008 to 2022

Variables (*N* = 34,955)	Depressive symptoms (CESD ≥ 4)			
	Male		Female	
	OR	95% CI	OR	95% CI
**Changes in parent–child relationships**				
No → No	1.00		1.00	
Yes → No	1.27	(0.89—1.81)	1.17	(0.83—1.65)
**No → Yes**	**0.63**	**(0.47—0.83)**	**0.67**	**(0.51—0.88)**
**Yes → Yes**	**0.69**	**(0.53—0.88)**	**0.73**	**(0.57—0.92)**
Age				
45–59	1.00		1.00	
60–69	1.17	(0.99—1.38)	1.27	(1.11—1.44)
70–79	1.21	(1.00—1.47)	1.50	(1.30—1.73)
80 ~	1.24	(1.00—1.54)	1.71	(1.45—2.02)
Region				
Rural area	1.00		1.00	
Urban city	1.15	(1.00—1.32)	1.09	(0.98—1.21)
Education				
Middle school or below	1.04	(0.86—1.25)	0.69	(0.54—0.90)
High school	1.03	(0.85—1.24)	0.69	(0.52—0.90)
College or beyond	1.00		1.00	
Marital status				
Married	1.00			
Single	1.51	(1.27—1.79)	1.27	(1.15—1.41)
Household income				
Quartile 4	1.00			
Quartile 3	0.77	(0.64—0.93)	0.89	(0.77—1.02)
Quartile 2	0.72	(0.61—0.86)	0.87	(0.76—0.99)
Quartile 1	0.74	(0.63—0.86)	0.82	(0.73—0.93)
Employment status				
Employed	1.00		1.00	
Non-employed	1.62	(1.42—1.86)	1.28	(1.14—1.43)
Social relations				
Yes	1.00		1.00	
No	1.75	(1.52—2.02)	1.53	(1.36—1.71)
Physical activity				
Yes	1.00		1.00	
No	1.52	(1.35—1.70)	1.20	(1.09—1.32)
Smoking status				
Yes	0.77	(0.68—0.88)	1.41	(1.10—1.81)
No	1.00		1.00	
Alcohol intake				
Yes	1.00	(0.87—1.14)	0.93	(0.83—1.04)
No	1.00		1.00	
Health status				
Healthy	1.00		1.00	
Unhealthy	2.65	(2.35—2.98)	2.74	(2.51—3.00)
Living with children				
Yes	1.00		1.00	
No	1.15	(1.01—1.32)	1.08	(0.97—1.19)
Life satisfaction				
Good (> 70)	1.00		1.00	
Normal (50 >, < = 70)	1.14	(0.97—1.34)	1.19	(1.05—1.36)
Bad (< = 50)	2.35	(2.00—2.76)	2.56	(2.25—2.91)
Before depression				
Yes	1.62	(1.42—1.85)	1.56	(1.41—1.72)
No	1.00		1.00	

Table 3 presents the results of the mixed-effects logistic regression subgroup analysis stratified by frequency and intensity of parent–child relationship, with adjustment for educational level, household income, marital status, employment status, social relations, physical activity, health status, and life satisfaction. Among men, maintaining or increasing contact frequency to at least once per month reduced the odds of depression (no change or increase: OR = 0.61; 95% CI = 0.47–0.78; decrease: OR = 0.67; 95% CI = 0.51–0.89). Among women, maintaining or achieving monthly contact also reduced the odds of depression (OR = 0.64; 95% CI = 0.50–0.81). Regarding meeting frequency, men with 3–6 or more meetings per year showed reduced odds of depression regardless of change. For women, having 3–6 meetings annually was associated with a reduction in depression risk (OR = 0.62; 95% CI = 0.48–0.81), and this effect became pronounced when the meeting frequency increased over time. Regarding financial support, receiving 1 million KRW (~ 750 USD) or more reduced depression odds in both genders, regardless of changes in support. Transitioning from no financial support to any support, regardless of the amount, significantly reduced depression in both men (OR = 0.70; 95% CI = 0.54–0.92) and women (OR = 0.73; 95% CI = 0.57–0.93). The results of the analysis, where the three factors were exclusively coded and combined, showed that compared to having none of the three factors, having all three factors present (men: OR = 0.63, 95% CI = 0.48–0.81; women: OR = 0.67, 95% CI = 0.53–0.85) and having both contact and meetings present (men: OR = 0.65, 95% CI = 0.50–0.84; women: OR = 0.70, 95% CI = 0.55–0.89) were associated with reduced odds of depression.

**Table 3:** Result of subgroup analysis stratified by interesting variables in 2006 to 2020

Variables	Depressive symptoms			
	Male		Female	
	OR	95% CI	OR	95% CI
Changes in frequency and intensity of relationships				
No → No (ref)	1.00		1.00	
Contact with children ^a^				
≥ **1/month → no change or increase**	**0.72**	**(0.54—0.95)**	**0.71**	**(0.55—0.92)**
≥ 1/month → decrease	0.67	(0.51—0.89)	0.92	(0.71—1.19)
Meeting with children				
3 ~ 6 times/year → no change	0.73	(0.56—0.95)	0.76	(0.60—0.97)
3 ~ 6 times/year → increase	0.62	(0.47—0.83)	0.62	(0.48—0.81)
3 ~ 6 times/year → decrease (no)	0.65	(0.45—0.94)	0.83	(0.61—1.14)
≥ 1/month → no change or increase	0.72	(0.54—0.95)	0.71	(0.55—0.92)
≥ 1/month → decrease	0.62	(0.47—0.82)	0.72	(0.56—0.93)
Financial support from children				
No → Yes	0.70	(0.54—0.92)	0.73	(0.57—0.93)
below 1 million KRW ^b^ → increase	0.58	(0.43—0.78)	0.71	(0.54—0.92)
> 1 million KRW → no change or increase	0.59	(0.44—0.78)	0.65	(0.50—0.83)
> 1 million KRW → decrease	0.62	(0.47—0.82)	0.64	(0.49—0.82)
Combination ^c^				
** Contact + meeting + financial support**	**0.63**	**(0.48—0.81)**	**0.67**	**(0.53—0.85)**
Contact + financial support	1.62	(0.64—4.13)	0.87	(0.44—1.70)
** Contact + meeting**	**0.65**	**(0.50—0.84)**	**0.70**	**(0.55—0.89)**
Financial support + meeting	1.12	(0.36—3.48)	2.79	(0.96—8.10)
Financial support	0.79	(0.53—1.18)	0.81	(0.58—1.14)
Contact	0.73	(0.39—1.37)	0.72	(0.43—1.22)
Meeting	0.47	(0.09—2.35)	1.44	(0.47—4.41)

^a^mean value adjusted for the number of children,

^b^median amount of financial support, ^c^ included no → yes, yes → yes Notes: adjusted for age, marital status, education level, income level, employment status, and health status

Table 4 presents the results of mixed-effects logistic regression subgroup analysis stratified by independent variables, with adjustment for educational level, household income, marital status, employment status, social relations, physical activity, health status, and life satisfaction. In both married men and women, relationship with children was associated with reduced odds of depression (men, no → yes: OR = 0.58, 95% CI = 0.42–0.80; women, no → yes: OR = 0.61, 95% CI = 0.44–0.86). Similarly, socially active men and women with parent–child relationship showed reduced odds of depression (men, no → yes: OR = 0.65, 95% CI = 0.48–0.88; women, no → yes: OR = 0.64, 95% CI = 0.48–0.85). Among physically active individuals and those who considered themselves healthy, a similar pattern was observed of reduced odds of depression in both men and women who maintained their relationship with children (men, no → yes: OR = 0.43, 95% CI = 0.26–0.72; women, no → yes: OR = 0.44, 95% CI = 0.28–0.70; men, no → yes: OR = 0.66, 95% CI = 0.47–0.93; women, no → yes: OR = 0.57, 95% CI = 0.40–0.80). The reduction in depression was stronger in the no → yes group than in the yes → yes group.

**Table 4:** Result of subgroup analysis by independent variables

Variables	Depressive symptoms						
	**No → No**	**Yes → No**		**No → Yes**		**Yes → Yes**	
	**OR**	**OR**	**95% CI**	**OR**	**95% CI**	**OR**	**95% CI**
**Male**							
**Marital status**							
Married	1.00	1.43	(0.95—2.13)	**0.58**	**(0.42—0.80)**	**0.69**	**(0.52—0.92)**
Single	1.00	0.95	(0.45—2.00)	0.95	(0.50—1.81)	0.65	(0.37—1.15)
**Social relations**							
Yes	1.00	1.30	(0.89—1.91)	**0.65**	**(0.48—0.88)**	**0.69**	**(0.53—0.89)**
No	1.00	1.27	(0.52—3.12)	0.61	(0.28—1.33)	0.90	(0.45—1.77)
**Physical activity**							
Yes	1.00	1.50	(0.84—2.69)	**0.43**	**(0.26—0.72)**	**0.53**	**(0.35—0.82)**
No	1.00	1.16	(0.74—1.81)	0.76	(0.54—1.08)	0.78	(0.58—1.06)
**Health status**							
Healthy	1.00	1.30	(0.85—1.98)	**0.66**	**(0.47—0.93)**	**0.69**	**(0.51—0.93)**
Unhealthy	1.00	1.23	(0.64—2.37)	0.59	(0.35—1.01)	0.71	(0.44—1.15)
**Female**							
**Marital status**							
Married	1.00	1.21	(0.79—1.86)	**0.61**	**(0.44—0.86)**	**0.72**	**(0.54—0.96)**
Single	1.00	1.12	(0.63—1.98)	0.82	(0.51—1.34)	0.79	(0.52—1.20)
**Social relations**							
Yes	1.00	1.13	(0.78—1.63)	**0.64**	**(0.48—0.85)**	**0.72**	**(0.56—0.92)**
No	1.00	1.40	(0.56—3.48)	1.02	(0.47—2.23)	0.81	(0.42—1.55)
**Physical activity**							
Yes	1.00	0.99	(0.56—1.74)	**0.44**	**(0.28—0.70)**	**0.51**	**(0.35—0.76)**
No	1.00	1.24	(0.83—1.87)	0.84	(0.60—1.16)	0.87	(0.66—1.15)
**Health status**							
Healthy	1.00	1.26	(0.83—1.91)	**0.57**	**(0.40—0.80)**	**0.73**	**(0.55—0.97)**
Unhealthy	1.00	1.04	(0.60—1.83)	0.89	(0.57—1.40)	0.79	(0.54—1.18)

Adjusted for age, marital status, education level, income level, employment status, and health status

## Discussion

In this study, we analyzed the relationship between parent–child interactions and parental depression using data from the KLoSA. Building on previous studies that primarily relied on cross-sectional designs and focused on a single dimension [20, 21], such as contact frequency or financial support, the present study employed a longitudinal design to examine the temporal changes and multidimensional combinations of associational and contact solidarity components. The analysis revealed that maintaining at least two forms of interaction—such as contact, meetings, or financial support—over a two-year period was significantly associated with reduced depressive symptoms in both men and women.

Maintaining at least two forms of contact (contact, meetings, or financial support) with children was found to reduce the risk of depression in older adults. In both older men and women, maintaining or increasing contact frequency (at least once per month), meeting 3–6 times per year, and receiving financial support were associated with a reduced risk of depression.

Further, maintaining contact and meetings with children as well as their frequency were associated with a reduction in depression among older adults [15, 20, 25, 26]. This finding supports the findings of existing Korean studies where associational solidarity with children, measured through communication and meetings, was found to reduce depression in aging parents [25]. A cross-sectional study in China demonstrated a dose–response relationship between contact frequency with children and the reduction of depression in older adults (measured using CES-D- 10) [15].

In this study, we examined the relationship between the frequency or intensity and combinations of these elements in child-parent relationships. Contact with children at least once a month (including once per week) was associated with a reduction in depression. An increase in contact frequency with children was associated with reduction in depression and improvement in life satisfaction among older adults. In a previous study, less frequent contact (< once a week) and meeting less than once monthly were found to increase the risk of depression in older adults [27]. Compared to these findings, the results of our study should be interpreted with a time-based perspective, emphasizing that for individuals with no prior contact, having at least monthly contact or even 3–6 meetings per year is meaningful. We included life satisfaction as a covariate and found that the reduction in depression remained significant after controlling for life satisfaction. Regarding meetings, maintaining or increasing the number of meetings to at least 3–6 times per year was associated with a reduction in depression. However, for women, the increase in the frequency of meetings was particularly important for reducing depression. In this regard, weekly visits by children were critical in reducing depression in aging parents [28].

Financial support was associated with a reduction in depressive symptoms, particularly when there was an increase in support over time. Existing literature presents diverse findings regarding financial support from children to parents [29–31]. Financial support has been shown to reduce parental depression in both the short and long terms, with an improvement in psychological well-being as the amount of financial support from children increases [29]. However, receiving financial support alone does not appear to affect depression, while providing financial support to children has been linked to a reduction in depressive symptoms [30]. High levels of depression were observed in men receiving financial support and women providing financial support [31]. The median amount of financial support in this data, 1 million KRW (approximately 750 USD) per year, may not always represent a substantial sum. For parents with significant financial need, receiving larger amounts of support could potentially increase depression. Therefore, regardless of the amount, financial support can be understood as a sign of filial piety, indicating that parents perceive their relationship with their children as positive and meaningful.

Furthermore, although contact, meetings, and financial support may not hold equal value, the combination analysis with mutually exclusive coding of their presence revealed that having all three forms of relationships or maintaining both contact and meetings was associated with a reduction in depression. This highlights the importance of not only material support but also emotional support in alleviating depressive symptoms among older adults. Compared to an increase in the amount of material support, an increase in contact has been associated with significantly higher parental satisfaction. This finding aligns with previous studies emphasizing the importance of emotional support, particularly in the context of improving parental financial stability [32].

The social network of older adults significantly influences their physical and psychological well-being [33]. Children living separately are often the most valuable part of the social network of old adults. As individuals age, contact with children provides emotional stability and a sense of being cared for, and a lack or reduction in such contact can negatively affect their mental well-being [34].

The present study has some limitations. First, while panel data allowed for the analysis of the association between changes in parent–child contact and depressive symptoms over time, the retrospective study design limits causal inferences. Although depressive symptoms in earlier waves were included as a covariate to account for potential reverse causality, residual confounding cannot be ruled out entirely. Additionally, unmeasured confounders, such as personality traits or environmental influences, may have affected the results. Therefore, these findings should be interpreted with caution, and future prospective studies are required to further validate these associations. Second, excluding participants who did not respond to depression-related questions may have introduced bias. Additionally, listwise deletion maintains data consistency but reduces the sample size, potentially limiting generalizability. These limitations should be carefully considered when interpreting the findings. Third, depression in this dataset was measured using the Korean version of the CES-D 10, a screening tool for depressive symptoms that is not intended for clinical diagnosis [35]. As such, it neither provides a clinical diagnosis nor accounts for biological risk factors. Additionally, there was a lack of consistency in the measurement tools across waves. Waves 1–4 employed the Anderson form, which includes only 10 items from the CES-D 20 [36], whereas wave 5 onward used the Boston form of the CES-D 10 [37]. To partially address this inconsistency, depression status was dichotomized using a cutoff score of 4 or higher. Although some prior studies have used a cutoff of 3 [38, 39], we opted for four to follow a more robust approach with a higher threshold to false positives. Although this method might have mitigated measurement inconsistencies, some variability may persist. Fourth, as this study assessed parent–child relationships based on parental responses, it has limitations in the interpretation of findings from a bidirectional interaction perspective and did not account for relationship quality. When parent–child relationship quality is high, the significance of meeting frequency, phone calls, and financial support may diminish. However, overall life satisfaction was included to partially address this limitation. Future research should incorporate relationship quality and a bidirectional perspective for a more comprehensive analysis. Fifth, as this study focuses on older adults in Korea, caution should be exercised when generalizing the findings to non-Confucian cultural contexts. In Confucian societies, strong expectations of emotional and financial support in parent–child relationships may have a greater impact on depressive symptoms than in cultures that emphasize individualism and economic independence. In Western societies, parent–child contact and financial support are often considered optional rather than obligatory, yet they still play a significant role in providing emotional and psychological support. Additionally, differences in socioeconomic structures and welfare systems may influence the applicability of these findings across cultures.

Despite these limitations, this study has several strengths. First, we used the KLoSA, which represents middle-aged and older populations in South Korea, and employed a longitudinal study design. We employed mixed-effects models that allowed us to reflect both within-person changes over time and between-person differences, providing precise and reliable results. Second, this study, which was conducted based on the solidarity theory, involved a multidimensional analysis of parent–child relationships, taking into account not only the time-based changes in the components of associational and contact solidarity but also the combinations and frequency of interactions. Third, we utilized a well-established depression screening tool (CES-D 10), which has proven reliability and validity.

## Conclusions

In this study, we found that regular and frequent contact with children, as well as financial support, were associated with a reduction in depressive symptoms among older adults. Moreover, maintaining these three forms of interaction at a certain level and an increased frequency of interactions was linked to improvements in mental health. These findings indicate the importance of emotional relationships and material resources in supporting the mental health of older adults.

## Supplementary Information

Supplementary Material 1.

Supplementary Material 2.

## Data Availability

https://survey.keis.or.kr/eng/index.jsp.

## Abbreviations

OECD: Orgainsation for Economic Cooperation and Development
CES-D 10: The Center for Epidemiological Studies Depression Scale 10 items
KLoSA: Korean Longitudinal Study of Aging
