# Supplementary material for: Impact of late parent–child relationship changes on parental depression: a longitudinal aging panel study
Source: BMC Public Health. 2025 Apr 15;25:1408. doi: 10.1186/s12889-025-22516-7 (PMC11998193; doi:10.1186/s12889-025-22516-7)
Supplement: Supplementary file 1 — Supplementary Material 1. [file 12889_2025_22516_MOESM1_ESM.docx]

Suppl 1. Flow chart of the study participants displaying the exclusion.

KLoSA enrollees

N=13,661

KLoSA enrollees at the baseline year 2006

N=10,254

Excluded new panels who did not participate in all waves from the 1^st^ to 8^th^ wave (n=3,407)

- 5^th^ wave new panels (n=920)

- 6^th^ wave new panels (n=890)

- 7^th^ wave new panels (n=830)

- 8^th^ wave new panels (n=767)

Excluded the initial year 2006, as change variables could not be generated (the state in 2006 was reflected in the change variable for 2008) (n= 4043)

Without children (n=261)

Missing value (n=1,474 )

Final study sample (N=4,476)

Men (n=1,719) and women (n=2,757)
