# Supplementary material for: Impact of late parent–child relationship changes on parental depression: a longitudinal aging panel study
Source: BMC Public Health. 2025 Apr 15;25:1408. doi: 10.1186/s12889-025-22516-7 (PMC11998193; doi:10.1186/s12889-025-22516-7)
Supplement: Supplementary file 2 — Supplementary Material 2. [file 12889_2025_22516_MOESM2_ESM.docx]

| Suppl 2. Result of subgroup analysis stratified by interesting variables in 2006 to 2020. | | | | | | |  |  |  |  |  |  |
| --- | --- | --- | --- | --- | --- | --- | --- | --- | --- | --- | --- | --- |
| **Variables** |  | **Depressive symptoms** | | | | | | | | | |  |
|  | **Male** | | | | |  |  | **Female** | | | |  |
|  | **OR** | | **95% CI** | | |  |  | **OR** | **95% CI** | | |  |
| **Changes in frequency and intensity of relationships** |  | |  |  |  |  |  |  |  |  |  |  |
| No → No (ref) | 1.00 | |  |  |  |  |  |  |  |  |  |  |
| **Frequency and intensity of the type of relationships** |  | |  |  |  |  |  |  |  |  |  |  |
| **contact with children** ^a^ |  | |  |  |  |  |  |  |  |  |  |  |
| No → No | 1.35 | | (0.24 | - | 7.52) |  |  | 2.61 | (0.46 | - | 14.70) |  |
| No → Yes | 0.99 | | (0.58 | - | 1.71) |  |  | 0.85 | (0.53 | - | 1.36) |  |
| 1–2 times per year → no change | 1.11 | | (0.20 | - | 6.10) |  |  | 5.81 | (1.22 | - | 27.62) |  |
| 1–2 times per year → increase | 1.15 | | (0.63 | - | 2.11) |  |  | 0.57 | (0.32 | - | 1.02) |  |
| 1–2 times per year → decrease (no) | 1.89 | | (0.41 | - | 8.64) |  |  | 0.90 | (0.20 | - | 4.03) |  |
| 3~6 times per year → no change | 1.09 | | (0.80 | - | 1.50) |  |  | 1.03 | (0.77 | - | 1.37) |  |
| 3~6 times per year → increase | 0.76 | | (0.57 | - | 1.01) |  |  | 0.83 | (0.64 | - | 1.07) |  |
| 3~6 times per year → decrease | 1.28 | | (0.70 | - | 2.35) |  |  | 1.47 | (0.83 | - | 2.61) |  |
| Once a 1 months or more frequently → no change or increase | 0.61 | | (0.47 | - | 0.78) |  |  | 0.64 | (0.50 | - | 0.81) |  |
| Once a 1 months or more frequently → decrease | 0.67 | | (0.51 | - | 0.89) |  |  | 0.92 | (0.71 | - | 1.19) |  |
| **meetings with children** |  | |  |  |  |  |  |  |  |  |  |  |
| No → No | 1.49 | | (0.64 | - | 3.46) |  |  | 0.73 | (0.34 | - | 1.54) |  |
| No → Yes | 0.74 | | (0.46 | - | 1.19) |  |  | 0.72 | (0.48 | - | 1.08) |  |
| 1–2 times per year → no change | 0.63 | | (0.35 | - | 1.13) |  |  | 0.75 | (0.48 | - | 1.18) |  |
| 1–2 times per year → increase | 0.60 | | (0.41 | - | 0.86) |  |  | 0.68 | (0.50 | - | 0.93) |  |
| 1–2 times per year → decrease (no) | 2.41 | | (0.98 | - | 5.96) |  |  | 1.15 | (0.50 | - | 2.65) |  |
| 3~6 times per year → no change | 0.73 | | (0.56 | - | 0.95) |  |  | 0.76 | (0.60 | - | 0.97) |  |
| 3~6 times per year → increase | 0.62 | | (0.47 | - | 0.83) |  |  | 0.62 | (0.48 | - | 0.81) |  |
| 3~6 times per year → decrease | 0.65 | | (0.45 | - | 0.94) |  |  | 0.83 | (0.61 | - | 1.14) |  |
| Once a 1 months or more frequently → no change or increase | 0.72 | | (0.54 | - | 0.95) |  |  | 0.71 | (0.55 | - | 0.92) |  |
| Once a 1 months or more frequently → decrease | 0.62 | | (0.47 | - | 0.82) |  |  | 0.72 | (0.56 | - | 0.93) |  |
| **financial support from children** |  | |  |  |  |  |  |  |  |  |  |  |
| No → No | 0.83 | | (0.64 | - | 1.08) |  |  | 0.86 | (0.67 | - | 1.10) |  |
| No → Yes | 0.70 | | (0.54 | - | 0.92) |  |  | 0.73 | (0.57 | - | 0.93) |  |
| below 1 million KRW ^b^ → no change | 0.79 | | (0.60 | - | 1.04) |  |  | 0.84 | (0.65 | - | 1.09) |  |
| below 1 million KRW → increase | 0.58 | | (0.43 | - | 0.78) |  |  | 0.71 | (0.54 | - | 0.92) |  |
| below 1 million KRW → decrease (no) | 0.73 | | (0.55 | - | 0.98) |  |  | 0.80 | (0.62 | - | 1.05) |  |
| > 1 million KRW → no change or increase | 0.59 | | (0.44 | - | 0.78) |  |  | 0.65 | (0.50 | - | 0.83) |  |
| > 1 million KRW → decrease | 0.62 | | (0.47 | - | 0.82) |  |  | 0.64 | (0.49 | - | 0.82) |  |

^a^ mean value adjusted for the number of children,

^b^ median amount of financial support
